# Fractal Dimension Analysis of Earth Magnetic Field during 26 August 2018 Geomagnetic Storm

**DOI:** 10.3390/e24050699

**Published:** 2022-05-14

**Authors:** Anna Wawrzaszek, Renata Modzelewska, Agata Krasińska, Agnieszka Gil, Vasile Glavan

**Affiliations:** 1 Space Research Centre, Polish Academy of Sciences, Bartycka Str. 18A, 00-716 Warsaw, Poland; akrasinska@cbk.waw.pl (A.K.); gila@uph.edu.pl (A.G.); 2 Faculty of Exact and Natural Sciences, Institute of Mathematics, Siedlce University, Konarskiego 2, 08-110 Siedlce, Poland; renatam@uph.edu.pl (R.M.); vasile.glavan@uph.edu.pl (V.G.)

**Keywords:** fractal dimension, time series, geomagnetic field, geomagnetic storms

## Abstract

We analyse the fractal nature of geomagnetic field northward and eastward horizontal components with 1 min resolution measured by the four stations Belsk, Hel, Sodankylä and Hornsund during the period of 22 August–1 September, when the 26 August 2018 geomagnetic storm appeared. To reveal and to quantitatively describe the fractal scaling of the considered data, three selected methods, structure function scaling, Higuchi, and detrended fluctuation analysis are applied. The obtained results show temporal variation of the fractal dimension of geomagnetic field components, revealing differences between their irregularity (complexity). The values of fractal dimension seem to be sensitive to the physical conditions connected with the interplanetary shock, the coronal mass ejection, the corotating interaction region, and the high-speed stream passage during the storm development. Especially, just after interplanetary shock occurrence, a decrease in the fractal dimension for all stations is observed, not straightforwardly visible in the geomagnetic field components data.

## 1. Introduction

Fractal-based analysis of time series has found extensive applications in various disciplines, also related to space weather aspects e.g., [1,2,3,4,5,6]. The characteristics most commonly used are the Hurst exponent, *H*, and the fractal dimension, $D_{F}$. The fractal dimension with $D_{F}\in \left[n,n+1\right)$ for a surface in *n*-dimensional space describes the roughness (complexity) of this set. The Hurst exponent of a time series (n=1) is associated with power-law correlations and describes persistence (for 0.5<H<1) and anti-persistence (when 0<H<0.5). The fractal dimension and the Hurst coefficient seem to be independent of each other: the fractal dimension is a local property, and long-memory dependence is a global characteristic [7]. However, by the assumption of statistical self-affinity, stationarity and absence of heavy-tails, the linear equation

(1) $$D_{F}=n+1-H$$

can be applied [1]. It should be noted that comprehensive studies confirmed that the linear relation (1) is warranted for a large number of real-world data sets. In particular, studies performed in [8,9] showed that geomagnetic storms exhibit statistical self-affinity properties.

Various methods can be applied to determine the Hurst exponent *H* or the fractal dimension $D_{F}$ from the analysed data [10]. Exampled methods that directly estimate the $D_{F}$ of the time series profile are Katz algorithm [11] or Higuchi method (HG) [12]. There is a large class of methods which focus on long-range correlations (and hence estimate *H*). For example, the power spectrum analysis (PSA) and the existence of a power law behaviour with a spectral exponent β allows to determine the fractal dimension by using the relation $H=2-D_{F}=\left(\beta \pm 1\right)/2$ [1]. Other methods which have been proposed to estimate *H* include: rescaled Hurst interval analysis (R/S) [13], rescaled range analysis (RRA) [14], detrended fluctuation analysis (DFA) [15] or its modification, named robust detrended fluctuation analysis (r-DFA) [16], wavelet-based analysis [17], structure functions (SF) [18], and also detrending moving average (DMA) [19,20]. It is worth also mentioning other descriptors, in particular Tsallis entropic measures [21], permutation entropy analysis [22], or phase space based dimensions [23], which fully characterize independent nonlinear characteristics.

In the context of geomagnetic field analysis, many of the mentioned methods have already been applied to describe the fractal nature of experimental data measured during various space weather conditions.

Among the first indications of the dynamical changes in the fractal features during geomagnetically disturbed periods were the results of Uritsky and Pudovkin described in [24]. In particular, the authors used the Fourier power spectra and data for the period 1973–1974 to show that the fractal dimension of AE-index fluctuations decreases sharply at the active period of disturbance and significantly increases at the end of the recovery phase.

Wanliss [8] applied DFA to the Sym-H geomagnetic storm index for the period 1981–2002. The author considered a large amount of data, identified the scaling range from 16 to 1024 min, and presented significant differences between the scaling exponents *H* for quiet and active intervals (periods when geomagnetic storms occurred). In particular, it was shown that for active intervals the scaling exponent is larger than 0.5 ($D_{F}<1.5$), indicating greater correlation and suggesting the organising power of storms.

Balasis et al. [9] focused on the fractal spectral properties of the average of low-latitude geomagnetic perturbations, measured in terms of the Dst-index. More precisely, using wavelet analysis methods, the authors performed a systematic analysis of the hourly-resolution Dst data registered during the whole year 2001, when two intensive storms appeared. The authors revealed that in the range of scales 2–128 h, the complexity (measured by fractal dimension) of fluctuations of the Earth’s magnetic field expressed via the Dst-index, decreases during intense magnetic storm periods.

Hamid et al. [25] performed a systematic analysis of the fractal properties of geomagnetic horizontal component data, H (to distinguish in our article the horizontal geomagnetic field traditionally denoted with the same letter H as the Hurst exponent, the latter one is written in italics), registered with 1s resolution by the low-latitude stations of Cebu and Davao in the Philippines, during quiet and active periods in August 2005 (medium solar activity level). The horizontal geomagnetic field component H $=\sqrt{B_{X}^{2}+B_{Y}^{2}}$ is determined by $B_{X}$ and $B_{Y}$, in the north and east directions. Applying three different fractal methods (PSA, RRA, and DFA), the authors identified the scaling range from 10 min to 6 h and determined Hurst exponents as 0.3–0.5 for quiet periods and 0.5–0.7 for active periods for both stations.

Zaourar et al. [26] explored the fluctuations of the horizontal component of the Earth’s magnetic field recorded by two INTERMAGNET observatories during the Solar Cycle 23 (1996–2005). To analyse multi-scale fractal properties of data, the wavelet-based approach has been used. Based on the analysis of six intense magnetic storms the authors suggested that a relatively sudden change related to the emergence of persistency of the fractal power exponent fluctuations precedes an intense magnetic storm. However, "preceding" means here that a reaction can be visible (shortly) after the initial arrival of the interplanetary shock, while the actual storm with its peculiar fractal characteristics develops only with some delay.

Hall [27] analyzed 25-year measurements, in the period 1988–2013, of the horizontal geomagnetic field from Tromsø (with 10 s resolution), using DFA and spectral analysis, taking into account all scales between 1 min and 1 day, and showed that the generalised Hurst exponent demonstrates overall anti-persistent character.

Nasuddin et al. [28] characterized H-component data during the quiet and disturbed days of 2011 using solely a single method (PSA). Data from 15 stations located at a wide range of geographic latitudes (region inside and outside the South Atlantic Anomaly (SAA)) were considered. The authors showed that the SAA region has a tendency to exhibits persistent fluctuations during both periods and related this finding to the Earth’s magnetic field strength. Moreover, it was found that as the Earth’s magnetic field strength increases (in high-latitude regions) anti-persistent behaviour was found.

Donner et al. [29] studied the temporal organisation of fluctuations inside the Earth’s magnetosphere using Dst-index and reported distinctive difference between quiet periods and geomagnetic storms. They found that ‘storm periods exhibit an elevated degree of dynamical regularity related to gradual trends of the Dst-index during the emergence of magnetic storms and the subsequent recovery phase’.

Alberti et al. [30] applied empirical mode decomposition and recurrence analysis (RA) to the Sym-H index. They considered a quiet period corresponding to the time interval between 1 and 10 August 2018 and a disturbed storm period between 24 August and 3 September 2018. The results showed that a scale-dependent dynamical transition occurs when moving from short (<200 min) to long (>200 min) timescales, with the more dynamical anomalies found in the behavior of the former. Furthermore, the authors confirmed that the fluctuations during a quiet period are more complex than during disturbed periods.

Recently, Rifqi et al. [31] investigated the geomagnetic H-component from two geomagnetic equatorial region stations in Southeast Asia. The authors considered the years 2009, 2013, and 2015 of low, intermediate, and high levels of solar activity, respectively. The r-DFA method was compared with other methods such as RRA, PSA, and DFA. Basing on the performed comparisons the authors recommended the DFA as the best one to characterise the fractal behaviour of geomagnetic data. Moreover, their findings report a higher level of Hurst exponent for disturbed days (affected predominantly by the occurrence of a geomagnetic storm) compared to quiet days.

Gil et al. [32] considered geomagnetic field components and modelled geoelectric field for the Belsk mid-latitude station during the full solar magnetic cycle of 1996–2019. Katz fractal dimension technique was applied in moving windows of 60 min and a significant increase in roughness (Katz dimension) of the analysed characteristics during severe geomagnetic storms was revealed. The authors underlined, however, that for quiet times, the Katz fractal dimension strongly underestimates the fractal dimension and it should be treated as a storm classifier rather than as a fractal dimension’s real value estimator.

This brief review shows that most studies have been devoted to analyse the geomagnetic indices rather than to study direct local properties of particular geomagnetic components on the Earth’s surface. Therefore, the objective of this work is to fill this gap and to perform for the first time a systematic and comparative analysis of the fractal dimension estimators as a proxy for complexity in particular for the horizontal geomagnetic field components registered by the four stations Belsk, Hel, Sodankylä and Hornsund at various latitudes from $52^{\circ }$ N to $77^{\circ }$ N during the period of 22 August–1 September, when the 26 August 2018 geomagnetic storm appeared. It should be noted that this strongest storm of 2018 has been extensively analysed from a physical point of view [33,34,35,36], while the fractal descriptors of complexity for this period have not yet been considered. To identify the fractal scaling and to compute the fractal dimension, we applied and compared three methods, SF, HG and DFA, the comparison of which in the context of geomagnetic data, to the best of our knowledge, has not been performed yet. The obtained results show temporal variation of the fractal dimension of geomagnetic field components, revealing differences between estimators and between field components. Moreover, we find a sensitivity of fractal dimension values to the change of physical conditions (appearance of the interplanetary (IP) shock, the coronal mass ejection (CME), the corotating interaction region (CIR) and the high-speed stream (HSS) passage around the storm), not obviously visible in the original geomagnetic data.

## 2. Data

### 2.1. Synthetic Fractal Time Series

To perform a comparison and selection of $D_{F}$ estimators for the analysis of geomagnetic field data, we prepared synthetic fractal time series. More precisely, we considered the Weierstrass cosine function (WCF), Takagi function (TF), and fractional Brownian motion (FBM), widely used in literature e.g., [37,38].

**Figure 1:**
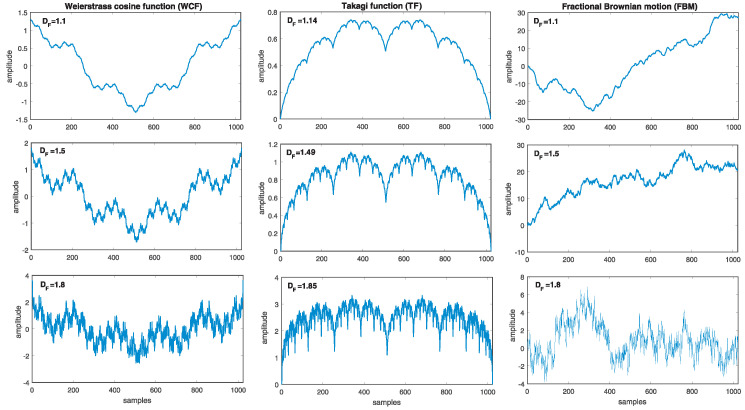
The Synthetic fractal time series: (first column) Weierstrass cosine function, (second column) Takagi function and (third column) fractional Brownian motion. The length of the presented data is N=1024 samples.

For the purpose of generating Weierstrass cosine function-based time series, we applied the relation:

(2) $$W\left(t\right)=\sum_{k=0}^{\infty }\gamma^{k(D_{F}-2)}\cos\left(2\pi \gamma^{k}t\right),$$

where $D_{F}$ is the fractal dimension of the generated signal for $1<D_{F}<2$ (0<H<1) and γ>1. The function WCF is continuous, nowhere differentiable, periodic for γ∈Z. In the study presented here, we assumed γ=5 and prepared WCF with fractal dimensions ranging from $D_{F}=1.1$ to $D_{F}=1.9$ with step 0.1. The first column of Figure 1 presents WCF generated for $D_{F}=1.1$, 1.5 and 1.8.

The Takagi function is expressed by the relation:

(3) $$K\left(t\right)=\sum_{k=0}^{\infty }a^{k}\phi \left(b^{k}t\right),$$

where ϕ(z)=z−⌊z⌋ and ⌊z⌋ denotes integer part of *z*, a∈[0,1], b∈Z and b>1. The TF for ab≥1 is everywhere continuous but nowhere differentiable. The K(t) defined with 1/2<a<1 and t∈[0,1] has a dimension equal to $D_{F}=\frac{\log(4a)}{\log(b)}$. Using the fixed value of parameter b=2 and for each of the chosen values of *a* varying from 0.55 to 0.95 with step 0.05, nine synthetic TF time series were prepared for further study. Three examples of the Takagi function generated for $D_{F}=1.14$ (a=0.55), $D_{F}=1.49$ (a=0.70) and $D_{F}=1.85$ (a=0.90) are shown in the middle column of Figure 1.

The fractional Brownian motion, introduced by Mandelbrot and Ness [39], is a non-stationary and self-similar stochastic process with Hurst exponent 0<H<1. Fractional Brownian motion FBM(t), being the generalisation of the classical Brownian motion BM(t) with H=0.5, is a moving average of increments dBM(t), in which past increments of BM(t) are weighted by the kernel ${\left(\Delta t\right)}^{H-0.5}$ and the variance of the increments is given by:

(4) $$Var\left(\Delta FBM\left(t\right)\right)=v{\left|\Delta t\right|}^{2H},$$

where *v* is a positive constant. We have generated FBM waveforms using the MatLab function wfbm(H,N) with the length *N* and Hurst exponent *H* corresponding to fractal dimensions from $D_{F}=1.1$ to $D_{F}=1.9$ with step 0.1. The third column of Figure 1 presents FBM generated for $D_{F}=1.1$, 1.5 and 1.8.

It is worth mentioning that some works e.g., [26] suggest that the dynamics of the geomagnetic field time series is similar to that of a fractional Brownian motion. Therefore, the FBM seems to be particularly useful in verifying the validity of $D_{F}$ estimators.

### 2.2. Geomagnetic Data

**Figure 2:**
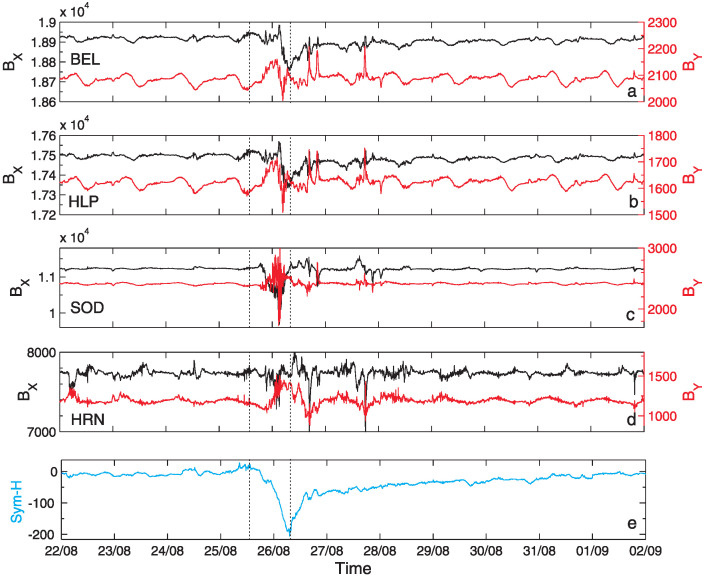
The Geomagnetic field components $B_{X}$ (black line) and $B_{Y}$ (red line) measured in nT by (**a**) BEL, (**b**) HLP, (**c**) SOD and (**d**) HRN stations between 22 August and 1 September, 2018 (time step 1 min). Panel (**e**) presents values of the symmetric H component (Sym-H) index for the same period. Black dashed lines denote the main phase of the geomagnetic storm (at ground level) between 25 August 2018 at 13:55 UT and 26 August 2018 at 08:15 UT [33].

We considered here data with 1 min resolution of horizontal geomagnetic field components $B_{X}$ and $B_{Y}$, in the north X and east Y directions, respectively, registered at various latitudes (from $52^{\circ }$ N to $77^{\circ }$ N) by four magnetometers listed in Table 1: Belsk (BEL), Hel (HLP), Sodankylä (SOD) and Hornsund (HRN), that are part of the INTERMAGNET network (International Real-time Magnetic Observatory Network). These two components $B_{X}$ and $B_{Y}$ play a key role in geomagnetic storms studies. We focused on the period 22 August–1 September, when the 26 August 2018 geomagnetic storm appeared [33,35]. The mentioned data are shown in Figure 2 with both quiet and storm (active) intervals. One may observe, that at various latitudes, starting from the lowest (Figure 2a), up to the highest (Figure 2d), the development of the storm was of different intensity. Moreover, both horizontal geomagnetic field components manifest evident diurnal variability. Figure 2e presents the values of the ground-based geomagnetic index Sym-H. Sym-H is a measure of the intensity of the globally symmetric component of the equatorial electrojet. It describes the geomagnetic disturbance field in the mid-latitudes with 1 min resolution derived from the horizontal magnetic field e.g., [40]. The index Sym-H is calculated on the base of measurements, given by the stations [41] San Juan (geographic latitude: 18.11${}^{\circ }$ N), Alibag (18.638${}^{\circ }$ N), Honolulu (21.32${}^{\circ }$ N), Tucson (32.17${}^{\circ }$ N), Fredericksburg (38.2${}^{\circ }$ N), Boulder (40.13${}^{\circ }$ N), Urumqi (43.8${}^{\circ }$ N), Memambetsu (43.91${}^{\circ }$ N), Chambon-la-Foret (48.025${}^{\circ }$ N), Martin de Vivies (37.796${}^{\circ }$ S), and Hermanus (34.425${}^{\circ }$ S).

The large, negative spike in Sym-H visible in Figure 2e corresponds to the period of considered geomagnetic storm. The 26 August 2018 geomagnetic storm, which appeared at the end of Solar Cycle 24, in the literature, is described as very specific event e.g., [34,36]. It originated from a weak coronal mass ejection on 20 August, caught by the Large Angle and Spectrometric COronagraph (LASCO) instrument on the Solar and Heliospheric Observatory (SoHO) around 21:12 UT, characterised by the magnetic cloud observed in the Earth vicinity from ∼12:15 UT on August 25 till ∼10:00 UT on August 26 [33]. It was preceded by the emergence of a new active region NOAA 2720 and the occurrence of a series of low B flares, from B1 to B4.1. The CME reached the Earth on August 25 and in combination with CIR/HSS [35] caused a major gradually commenced storm, with minimum Sym-H index value of −205 nT (Figure 2e), Dst-index minimal value −174 nT [42] and maximum Kp = 7+ [43]. CIR impact on Earth started ∼10:00 UT on August 26 and lasted until the evening hours and the HSS lasted for several days, from ∼18:00 UT on August 26 until around noon on 30 August (compare Figure 2 in [33]).

This was a strong G3 geomagnetic storm according to NOAA (National Oceanic and Atmospheric Administration) scale [34]. During this storm, solar wind speed was rather low, increasing above 500 km/s at the recovery phase of the storm, plasma temperature was around 10${}^{5}$ K, the interplanetary electric field reached 7 mV/m. Around 06:00 UT, on 26 August, the heliospheric magnetic field (HMF) strength increased to 18.1 nT [35]. The HMF Bz component turned southward around 14 UT on 25 August, remaining negative for more than 17 h, with a minimal value of −16.8 nT [36]. Using the magnetic data of low and mid-latitudes a longitudinal asymmetry of the magnetic field variations was shown [44]. Analysis of the thermospheric neutral mass density revealed a growth of 300–500% compared with the quiet-time values [34]. During the recovery phase of this storm there were observed peaks in the particle count rates indicating a phenomenon of electrons being accelerated in the radiation belts.

## 3. Methodology

### 3.1. Fractal Methods Selection

There are several $D_{F}$ estimation approaches that can be used to describe and understand the scaling properties of experimental data and reveal fractal structure. These algorithms differ in terms of accuracy, sensitivity to noise, and dependency of the estimation on the selected length of the time window [38]. Moreover, some $D_{F}$ methods require preliminary adjustments of several sensitive parameters.

In this work, basing on the review of literature and continuing our previous studies [32,45], we initially compared a few methods: the Katz algorithm [11], which revealed discriminating power [32,38], its modifications [46,47], Higuchi method [12], Structure Function scaling [18,48,49,50], and Detrended Fluctuation Analysis [15,51].

**Figure 3:**
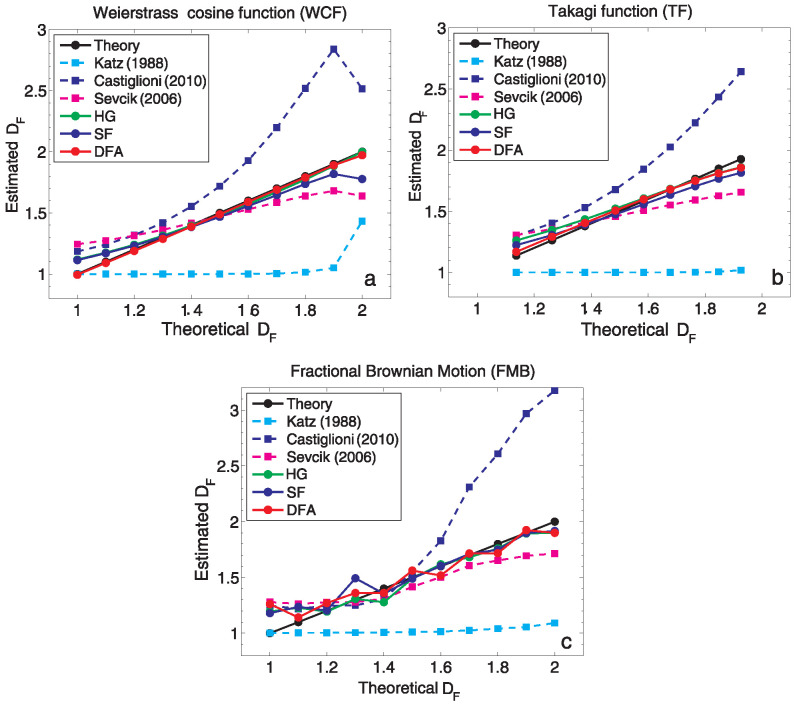
Estimated $D_{F}$ versus theoretical $D_{F}$ of synthetic fractal signals using Katz and its two modifications proposed by Sevcik [46] and Castiglioni [47], HG, SF, and DFA method, for (**a**) Weierstrass cosine function (WCF), (**b**) Takagi function (TF), (**c**) fractional Brownian motion (FBM). The number of samples in each of the considered signals is 1024.

Figure 3 presents the initial comparison of the methods mentioned. We see that Katz method underestimates the fractal dimension of synthetic (ideal) data, while one of its modification proposed by [47] overestimates the $D_{F}$. The analysis performed indicates that the other three selected methods, SF, HG and DFA, give the best comparable results. Therefore, in the remainder of this work only these three methods will be considered in detail, and applied to reveal and describe the fractal nature of geomagnetic field data. It is worth underlining that the HG technique allows for the direct estimation of the dimension $D_{F}$, while in the frame of indirect SF and DFA methods, first the persistence or anti-persistence of time series expressed by Hurst exponent is considered before Equation (1) is exploited. The application of Equation (1) requires the assumption of stationarity and the absence of heavy-tails (confirmed by us before the analysis).

### 3.2. Structure Function Scaling

The first technique that we adopted to evaluate the Hurst exponent is based on the scaling properties of the structure function (SF). This method investigates of the scaling properties of a time series directly via the computation of SF being the *q*th-order moments of the distribution of the increments over the time τ e.g., [18,48,49,50]:

(5) $$S\left(q,\tau \right)=\langle |X\left(t+\tau \right)-X\left(t\right){|}^{q}\rangle ,$$

where 〈〉 denotes the mean value. The *q*-th order moments of the distribution of the increments are good quantities to characterise the statistical evolution of a stochastic variable X(t). Hurst analysis examines whether some statistical properties of time series X(t) scale with time resolution and observation period *T*. The Hurst exponent H(q) can be defined from the scaling behaviour of S(q,τ) as:

(6) $$S\left(q,\tau \right)\propto \tau^{qH(q)}.$$

For q=1, H(1) describes the scaling behaviour of the absolute values of the increments. We are interested in the temporal profile of the Hurst exponent of geomagnetic field components with a resolution of one hour. Thus, we consider a moving window of 24 h with 1 min resolution of geomagnetic data, corresponding to time windows of 1440 min with a displacement of 1 h. The analysed data are detrended by removing the linear trend. Here, we consider the case for q=1 (hereafter, in this article we designate H(1)=H). The Hurst exponent *H* is computed through a linear least-squares fitting of the logarithm of relation (6).

### 3.3. Higuchi Method

The second technique, applied in the frame of the paper, is the Higuchi (HG) method [12,52], which analyses the fluctuations of the signal by investigating the defined length of the curve for different magnifications of the time axis of the signal. In the frame of this method, for the time series X(t) of size *N* several new time series are constructed by subsampling:

(7) $$X_{k}^{m}:X\left(m\right),X\left(m+k\right),X\left(m+2k\right),\ldots ,X\left(m+\left\lfloor \frac{N-m}{k}\right\rfloor \cdot k\right)$$

with m=1,2,…,k. Here *m* and *k* denote the initial time and time interval, respectively. The length of the curve $X_{k}^{m}$ is defined as follows:

(8) $$L^{m}\left(k\right)=\sum_{i=1}^{\left\lfloor \frac{N-m}{k}\right\rfloor }|X\left(m+ik\right)-X\left(m+\left(i-1\right)k\right)|\frac{N-1}{\left\lfloor \frac{N-m}{k}\right\rfloor \cdot k^{2}}.$$

Finally, the curve length over the time interval *k*, denoted by 〈L(k)〉, is defined as the average value of $L^{m}\left(k\right)$ over all *m*. If $\langle L\left(k\right)\rangle ∼k^{-D_{F}}$ then the curve is a fractal with fractal dimension $D_{F}$.

It is worth pointing out that before the analysis by using the HG method, it is necessary to determine how high should be the maximum *k* value ($k_{\max}$). Selected studies (e.g., [53]) recommend to compute the estimates for increasing values of *k* and the use the value where the estimates reach a plateau. In the presented study, basing on initial tests, we finally used $k_{\max}=N/10$.

### 3.4. Detrended Fluctuation Analysis

The third method, applied in the frame of this work, is so-called detrended fluctuation analysis (DFA) [15,51]. DFA methodology, whose predecessors were the rescaled Hurst interval analysis [13] and fluctuation analysis (FA) [14], has been proposed by Peng et al. [15], and from that time extensively used and developed [51,54]. In the first step of the DFA method, the time series *X* of size *N* is integrated by computing the accumulated departure from the mean of the whole series:

(9) $$Y\left(l\right)=\sum_{i=1}^{l}\left[X-\langle X\rangle \right]$$

where l=1,⋯,N and $\langle X\rangle =1/N\sum_{j=1}^{N}X\left(j\right)$. Next, the integrated series *Y* is divided into $N_{s}=\left\lfloor \frac{N}{s}\right\rfloor$ non-overlapping segments *v* (subseries) of length *s*. In the third step, the series *Y* is locally detrended. More precisely, for a given segment $v=1,\ldots ,N_{s}$ of size *s*, the characteristic size of the fluctuation *F* for the integrated and detrended series is calculated by

(10) $$F^{2}\left(v,s\right)=\frac{1}{s}\sum_{j=1}^{s}{\left[Y\left(\left(v-1\right)s+j\right)-Y_{v}^{p}\left(j\right)\right]}^{2}$$

where $Y_{v}^{p}\left(j\right)$ denotes an *p*-th order polynomial fitted to the *Y* in segment *v*. It is worth stressing that various degree polynomial functions can be used e.g., [51,55]. In the analysis presented here, similarly to SF methodology, the 1-st order (p=1) polynomial has been applied. The computation expressed by Equation (10) is repeated over various segment sizes *s* to provide a relationship between *F* and *s*:

(11) $$F\left(s\right)=\sqrt{\frac{1}{N_{s}}\sum_{v=1}^{N_{s}}\left[F^{2}\left(v,s\right)\right]}.$$

Finally, a power law is expressed as:

(12) $$F\left(s\right)\propto s^{\alpha },$$

where α denotes the scaling exponent expressed as the slope of a double logarithmic plot of F(s) as a function of *s*. For a stationary fractional Gaussian noise H=α, while for a non-stationary fractional Brownian motion, the Hurst exponent is H=α−1.

One of the important aspects of the procedure matching the exponent *H* (see relation (12)) is the appropriate consideration of the minimal and maximal segment size *s*. Selected studies recommend to use the maximal box size equal to one-tenth of the signal length (N/10) [55,56] or s>N/4[57]. This upper bound is related to the fact that for very large scale sizes, the function computed by Equation (11) ceases to be statistically significant as the number of segments $N_{s}$ decreases. Then, for too small scales, systematic fluctuations in the scaling factors may also occur. As reported in various studies e.g., [58], the minimum value of the scale parameter should not be smaller than 10 and generally larger than the order *p* of DFA.

### 3.5. Influence of Data Length

It is worth underlining that the fractal dimension of the fluctuations can ultimately depend on the width of the time windows used for computation e.g., [37,38]. Therefore, in this section, we verified the estimation accuracy of the SF, HG, and DFA based fractal dimension estimates using the three parametric fractal functions described in Section 2.1. Specifically, considering different lengths of synthetic data (N=128, N=256, N=512, N=1024, and N=2048) we tested the accuracy of fractal dimension determination using Root Mean Square Error (RMSE) and Mean Error (ME) for each method: $RMSE=\sqrt{\frac{1}{J}\sum_{M=1}^{J}{\left(D_{\mathrm{Fe}}-D_{\mathrm{Fth}}\right)}^{2}}$, $ME=\frac{1}{J}\sum_{M=1}^{J}\left(D_{\mathrm{Fe}}-D_{\mathrm{Fth}}\right)$ where, $D_{\mathrm{Fe}}$ and $D_{\mathrm{Fth}}$ are the estimated and theoretical fractal dimensions, respectively, *J* corresponds to the number of considered fractal dimension $D_{F}$ values from 1 to 2 for each synthetic function. A quantitative summary of the analyses is presented in Table 2 and Table 3, where RMSE and ME values provide an average distance between $D_{F}$ determined using SF, HG and DFA methods and the theoretical values of $D_{F}$.

The results in Table 2 and Table 3 show that the greatest differences between theoretical and computed $D_{F}$ values (the biggest RMSEs and MEs) are observed for short time series, namely, for N=128 or N=256. We see the general trend of the increase in accuracy with the increase in the length of considered time series *N*. On the other hand, the results are influenced by the type of synthetic fractal function considered. For FBM the situation seems to be less ordered and, unexpectedly, the accuracy of all three methods for N=512 is lower than those for shorter time series. Moreover, Table 2 and Table 3 reveal another important observation that from the time series length N=1024 the accuracy of determination of $D_{F}$ remains rather at the same level and the increase in the length of the signal does not improve the results. Taking this fact into account, in our further study related to fractal analysis of geomagnetic field components, we considered a moving-scale window of 24 h with 1 min resolution of geomagnetic data with the displacement of 1 h. Thus, for each case considered using SF, HG and DFA, we have analysed data in time windows of 1440 min (N=1440). Moreover, this choice of moving window allowed us to avoid the influence of 24-hour periodicity in geomagnetic field data. Therefore, the scaling features of the analyzed data concentrated in the time scales that do not exceed 24 h (for more details see Section 4 below).

## 4. Results and Discussion

### 4.1. Range of Fractal Scaling

Confirmation and determination of the proper range of fractal scaling remains crucial for appropriate methodologies application and related parameters determination. As we have underlined in Section 3, the methods selected by us have limitations with respect to the ranges of scales under consideration. For the SF method, the range of scales τ has an upper limitation, $\tau \leq \tau_{\max}$, where $\tau_{\max}=N/10$ is often applied. Similarly, for Higuchi’s method, only the maximum interval length $k_{\max}$ is optimised and then the range of scales considered is $1\leq k\leq k_{\max}$. For DFA, two parameters related to scale are evaluated, $s_{\min}\leq s\leq s_{\max}$, where $s_{\min}$ and $s_{\max}$ directly depend on the length of the signal and the order of DFA (see Section 3.4). Besides the application of the mentioned limitations, various systematic tests and visual inspections are still required to allow for proper identification of stable fitting ranges for each method.

**Figure 4:**
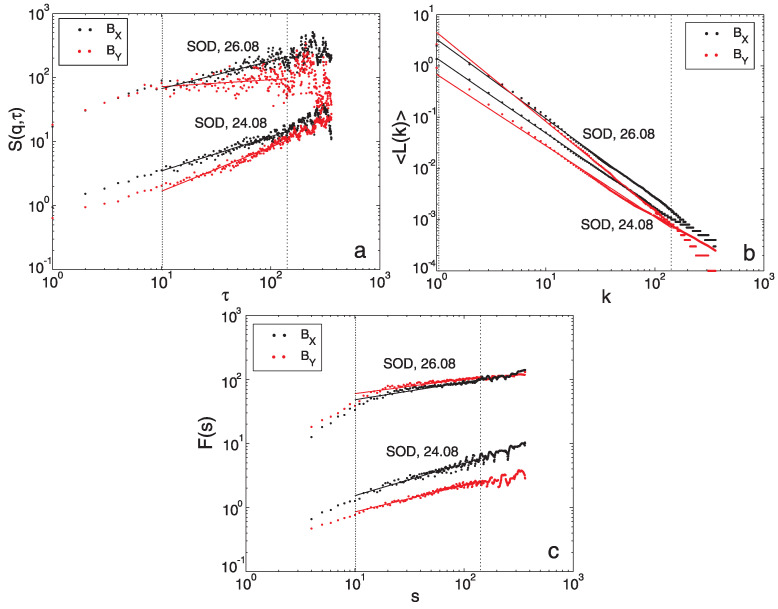
The Fractal scaling identified during the application of SF (**a**), HG (**b**) and DFA (**c**) methods for $D_{F}$ computation. Results for two geomagnetic field components $B_{X}$ (black) and $B_{Y}$ (red) measured by SOD station during quiet (24 August) and disturbed by storm (26 August) days are shown. The figure presents the results for scales from 1 to N/4=360 min while dashed lines indicate the identified scaling ranges.

Examples of logarithmic scaling plots that yielded the $B_{X}$ (black) and $B_{Y}$ (red) data measured by SOD station during a quiet day (24 August) and day disturbed by the storm (26 August), respectively are provided in Figure 4. We show the scaling for SOD station, as at for which the most significant variation of geomagnetic field components during the considered storm (see Figure 2) was revealed.

Each of the panels of Figure 4 shows the logarithmic scaling plots produced by applying the corresponding fractal analysis technique. Figure 4a presents different SF scaling for the days 24 August and 26 August revealing less ordered behaviour for the latter case. The identified common scaling range for both cases varies from 10 min to 144 min (≤*N*/10). Figure 4b, which corresponds to the HG technique, presents more ordered behaviour with wider range of scaling starting from 1 min to 144 min. It should be noted that some small deviation from the fitted solid line can be identified on shorter timescales, especially for $B_{Y}$ component. The Figure 4c, which is devoted to DFA method, again reveals differences in the nature of scaling for quiet (24 August) and disturbed by storm (26 August) days. In particular, we see change in scaling at a scale of 10 min. Finally, a reliable linear fitting, common for all cases considered during the determination of the fractal dimension by DFA method, has been identified in the scaling range from $s_{\min}=10$ min to $s_{\max}=144$ min.

The scaling ranges identified in the frame of this work are smaller than those considered in some previous studies devoted to analysis of geomagnetic indices (e.g., [8,9,59]). However, in the frame of this paper, we analyse much smaller, 1-day measurement windows. Moreover, our scaling range seems to agree with the time scales between 30 and 300 min considered by Alberti et al. [60] during the determination of the generalised fractal dimensions for the Sym-H index. Furthermore, Rifqi et al. [31], proposed the consideration of the scaling range from 10 min to 6 h for the analysis of the H-component, which seems to be in accordance with our observations.

It is worth underling indications that especially the lower frequency components (with time scales of several hours) of the magnetic field fluctuations cause a reduction in dimensionality during magnetic storms (see Figure 5 in [30]). The latter findings are consistent with previous results for the Dst-index (e.g., [29]). Taking this fact into account, we have performed an additional test and extended the range of scaling up to N/4=6 h, which for many cases (but not all) could also be selected. The results for this larger scaling range (not shown here) were comparable with the results obtained for N/10=144 min, with a small tendency towards smaller estimates of $D_{F}$.

### 4.2. Fractal Dimension of Geomagnetic Field Components

**Figure 5:**
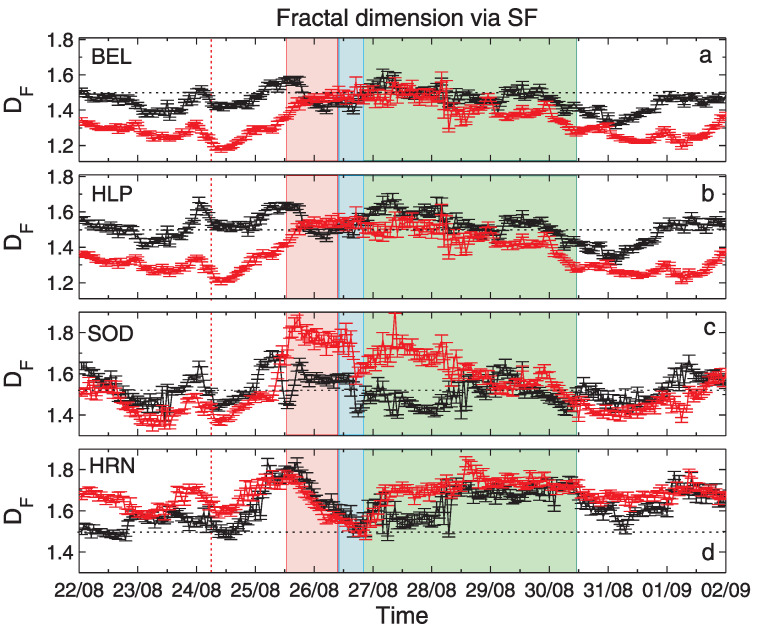
Values of the fractal dimension $D_{F}$ estimated for the geomagnetic field components, $B_{X}$ (black) and $B_{Y}$ (red) by using the structure function scaling technique. Particular panels present results for $B_{X}$ and $B_{Y}$ registered by stations: (**a**) BEL, (**b**) HLP, (**c**) SOD and (**d**) HRN.

**Figure 6:**
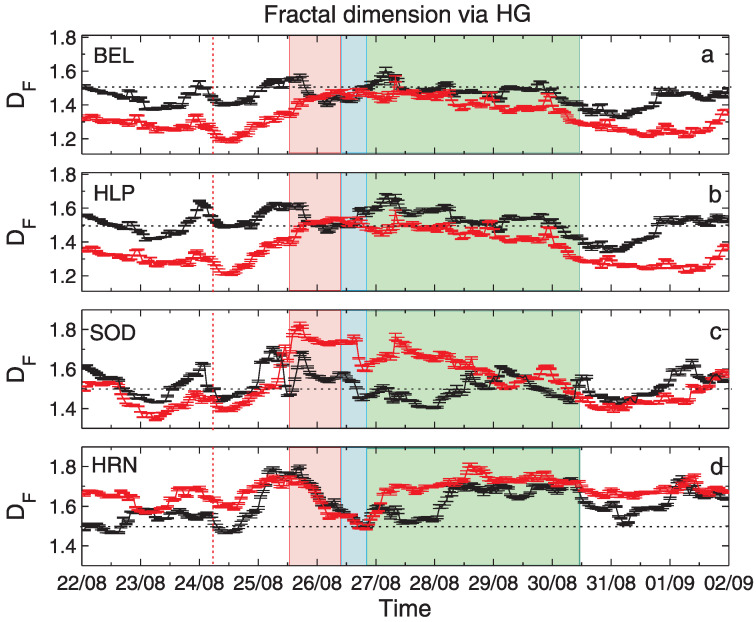
Values of fractal dimension $D_{F}$ computed for geomagnetic field components, $B_{X}$ (black) and $B_{Y}$ (red) by using the Higuchi method. Particular panels present results for $B_{X}$ and $B_{Y}$ registered by stations: (**a**) BEL, (**b**) HLP, (**c**) SOD and (**d**) HRN.

**Figure 7:**
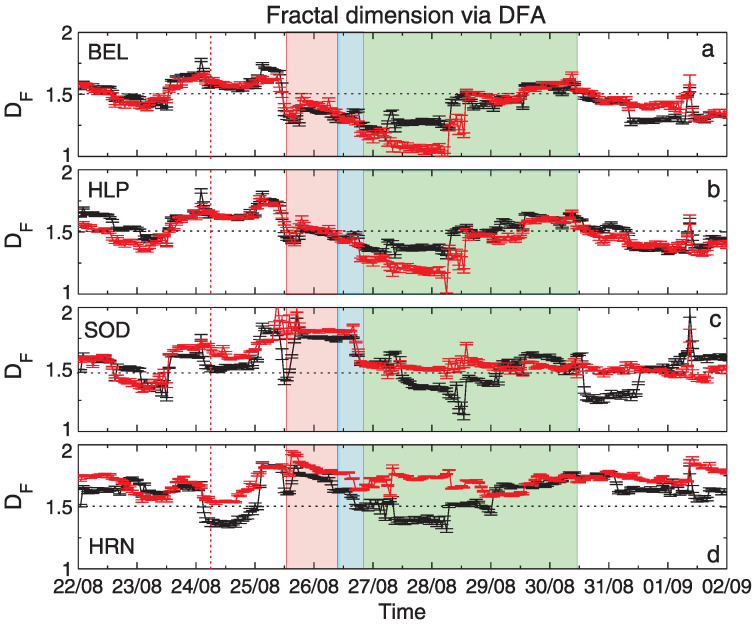
Values of fractal dimension $D_{F}$ estimated for geomagnetic field components, $B_{X}$ (black) and $B_{Y}$ (red) by using DFA. Particular panels present results for $B_{X}$ and $B_{Y}$ registered by stations: (**a**) BEL, (**b**) HLP, (**c**) SOD and (**d**) HRN.

The results of the application of the three fractal methods to geomagnetic data described in Section 2.2 are shown in Figure 5, Figure 6 and Figure 7. The vertical bars indicate the errors in the determination of $D_{F}$, as the accuracy of the regression line slope in the logarithmic scaling produced by applying the corresponding fractal analysis technique (see Section 4.1). From top to bottom, the panels show results for stations: BEL, HLP, SOD and HRN. For a better interpretation of the obtained results, some physical events related to the 26 August geomagnetic storm, discussed in Section 2.2, were additionally denoted in Figure 5, Figure 6 and Figure 7. More precisely, the red dashed line shows the moment of the arrival of an interplanetary shock, IP, the red shaded rectangle corresponds to the coronal mass ejection, CME, while the blue and green shaded regions show the corotating interaction region, CIR, and the high-speed stream, HSS, respectively. All these events are discussed in [33].

Figure 5 presents the temporal variation of $D_{F}$ for the geomagnetic field components, $B_{X}$ (black) and $B_{Y}$ (red), determined by the SF method. Figure 6 presents values of fractal dimension $D_{F}$ estimated by using the HG method. We can see almost perfect agreement between the SF and HG methods for both geomagnetic components $B_{X}$ and $B_{Y}$ for all stations. The fractal dimensions for the BEL and HLP stations show similar time variations. The same can be seen for the SOD station, but with larger amplitude of this variability. The fractal dimensions for both components coincide in the period 26 to 29 August 2018 and differ for the rest of the period for BEL and HLP. An opposite situation can be seen for SOD. The fractal dimensions of $B_{X}$ component oscillate around 1.5. Fractal dimensions of the $B_{Y}$ component start with values below 1.5, then increase to ∼1.5 for BEL and HLP and up to ∼1.8 for SOD during the onset of the storm period, further decrease is observed after 28 August 2018.

Now, we consider the time variation of $D_{F}$ on the basis of the physical conditions connected with IP shock, CME, CIR and HSS passage around the storm. Here we are rather concentrated on the methodological aspects, physics of this event was broadly discussed in [30,33]. The sharp increase in $D_{F}$ can be observed just before the IP shock in both geomagnetic components, for all stations, even though the IP shock moment is invisible in the temporal changes of the geomagnetic field data. During the CME passage, SOD $D_{F}$ shows rapid growth especially for the $B_{Y}$ component (see Figure 2a–d), for HRN this local maximum in $D_{F}$ is seen earlier at the boundary of the CME passage for both components, the HLP and BEL stations show a gradual increase in the $B_{Y}$ component and a decrease in $B_{X}$ component. During the CIR passage, SOD $D_{F}$ exhibits a rapid decrease for both components, also for HRN $D_{F}$ has a local minimum, HLP and BEL $D_{F}$s are rather stable ∼1.5. HSS period is associated with a gradual decrease in $D_{F}$ for HLP and BEL and for $B_{Y}$ for SOD, where HRN seems to have some rising trend for both components.

Let us now have a look at the results shown in Figure 7, obtained by applying the DFA methodology. Comparing the panels with results for four observatories, we see that the fractal dimension’s values estimated for SOD and HRN are higher than those obtained for BEL and HLP data. Please note that similar conclusions were received by using SF and HG techniques. It can state the confirmation of the fact that the nature and complexity of local geomagnetic field fluctuations are related to the geographic/geomagnetic position of the stations, more precisely to the strength of the Earth’s magnetic field, as underlined by, e.g., [28]. Moreover, Figure 7 shows that the fractal dimension of $B_{Y}$ for HRN station is contained in the interval [1.5, 2] exclusively, revealing mostly anti-persistent behaviour. In the remaining cases, the situation is more variable and we can identify periods when the complexity of $B_{X}$ and $B_{Y}$ increases ($D_{F}>1.5$) or when a decrease is observed. In particular, in contrast to the results presented in Figure 5 and Figure 6, we clearly see here a slow decrease in fractal dimension $D_{F}$ of geomagnetic field components. This decrease for BEL, HLP and HRN stations starts with the storm and CME (red shaded region), while for SOD station the decline appears later, with the CIR passage (blue shaded region).

Additionally, a comparison of Figure 7 with Figure 5 and Figure 6 reveal that in the case of DFA we do not observe similarly significant differences between the complexity of geomagnetic field components as identified by using SF and HG methods. The differences between the $D_{F}$ for $B_{X}$ and $B_{Y}$ appear only after CIR occurrence. Additionally, for SOD station (Figure 7c) after HSS passage we see rapid decrease in $D_{F}$ for $B_{X}$ not observed for $B_{Y}$.

The results in Figure 7 suggest that the fractal dimension estimated by using the DFA technique, similarly to SF and HG, "feels" the physical conditions related to IP shock, CME, CIR or HSS passage during the storm. Especially, just after IP shock’s occurrence (red dashed line), we see a decrease in $D_{F}$ for all stations, at the beginning of CME, a rapid and short-lasting reduction of $D_{F}$ is also observed. We can speculate that the discussed variation of the fractal dimension $D_{F}$ reflects changes in scaling of the $B_{X}$ and $B_{Y}$, thus can support further studies of the influence of mentioned physical phenomena on geomagnetic field.

### 4.3. Fractal Dimension of Sym-H

**Figure 8:**
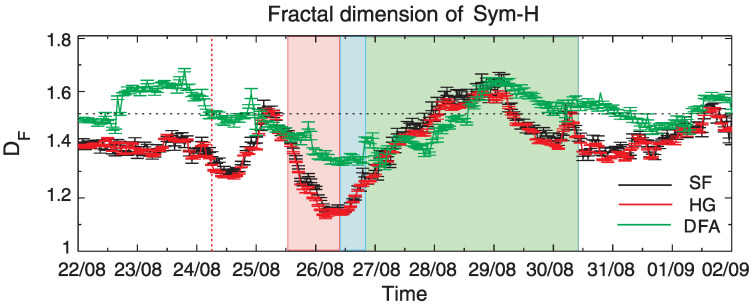
Values of fractal dimension $D_{F}$ estimated for the Sym-H index by using SF (black), HG (red) and DFA (green) methods.

For comparison, Figure 8 presents the values of fractal dimension $D_{F}$ of Sym-H index between 22 August and 1 September 2018.

As before, three methods, SF (black), HG (red) and DFA (green) were systematically applied. We observe here a clear decrease in $D_{F}$ (increase in *H*) during the considered 26 August 2018 geomagnetic storm, but the level of this change depends on the used method. We observe a more effective and more rapid reduction of $D_{F}$ during the geomagnetic storm period for SF and HG methods than for DFA. On the other hand, $D_{F}$ estimated by DFA presents higher values of fractal dimension with the tendency to be around 1.5 for most part of quiet periods.

Additionally, we conducted a test analysis (not presented in this paper) for the Dst-index in 2001 following the paper [29], and using our approach of SF, HG and DFA, confirms the decrease in $D_{F}$ during a period with two intense (G4) storms.

In general, for all applied methods, the storm conditions are found to have a smaller fractal dimension than for quiet times. This is in agreement with previous analysis devoted to the same geomagnetic features but different storms e.g., [29,61,62]. In particular we can mention the analysis of the Dst-index in 2001, during two intensive storms [29], as well as the study of another very well known storm in March 1989 [63]. These results are consistent with [21], where it is shown that the Tsallis entropy of the Dst time series decreases during intense magnetic storms (Dst <−150 nT).

All of these results support the findings that the complexity of geomagnetic field decrease during the storm as a result of a self-organizational character of the dynamics during geomagnetic storm e.g., [29].

We see, however, that conclusions for Sym-H seem to contradict some results for the geomagnetic field components presented in Figure 5 and Figure 6. It is worth noting that all the stations listed above (as a source for Sym-H) are located at lower latitudes than the stations used in our studies, thus the comparison with Sym-H features is not straightforward. Since Sym-H is demonstrative for the large-scale magnetospheric structures, but not very responsive to small-scale phenomena, it is impossible to pronounce all changes of the full environment. An additional reason for the discrepancy can be related to the fact, that the geomagnetic indices are obtained by the spatial averaging of local and more variable magnetic field data and this kind of operation can influence the fluctuation characteristics. Moreover, Alberti et al. [30] suggested the existence of different dynamical components characterizing the Sym-H index variability. One can raise the question, whether the same observations will be recovered by using local magnetic field components.

In light of the obtained differences between the results for Sym-H and geomagnetic field components it is worth also mentioning very recent studies performed in [23]. The authors, using a different technique based on local phase space dimension analysis, have considered two geomagnetic indices: Sym-H and AL, where AL is representative for high latitude regions and allows to describe the westward auroral electrojet. Alberti et al. [23] obtained, in general, that the number of degrees of freedom, required to describe the phase space dynamics, increases at high latitudes in contrast to a reduced number of degrees of freedom observed during geomagnetic storms at low latitudes. The authors suggested the existence of concurrent effects between high and low latitude current systems: the auroral activity is externally driven by the solar wind only, the enhancements of the equatorial activity during geomagnetic storm are related to both the solar wind and high latitude processes. Our results, which depend on the location of the stations whose data were analyzed, seem to confirm this observation. This is particularly evident for the higher latitude HRN and SOD stations in comparison to mid latitude BEL and HLP data, for which a different behaviour of $D_{F}$ is observed for all considered methods.

Finally, our results suggest that the considered methods are capable of indicating the relationship between the fractal dimension and the occurrence of interplanetary events and their influence on Earth magnetic field. However, the calculated fractal dimensions evolve in a unique way through the various stages of the analysed period around a geomagnetic storm for different methods. There does not seem to be one-to-one correspondence between SF-HG compared to DFA. In our opinion, this might be connected with different fluctuation treatment in time series by different methods. Especially the DFA method, which has already confirmed its usefulness in geomagnetic data analysis e.g., [8,31], seems to better deal with nonstationarity in time series. Moreover, Muñoz et al. [63] suggested that during the main phase of a magnetic storm, the level of multifractality is large, consistent with its more turbulent nature, comparing to an intermediate or even monofractal nature for quiet conditions. Growth of multifractal nature can be related to the occurrence of intermittent bursts connected with spatiotemporal turbulence in the equatorial plasma sheet regions and the impulsive energy-release phenomena [62]. This would also have a direct impact on $D_{F}$ values estimated via different methods.

## 5. Conclusions

We performed a systematic analysis of the fractal scaling of geomagnetic field components registered at four different geographic latitudes (from $52^{\circ }$ N to $77^{\circ }$ N) during the period 22 August–1 September, when the 26 August 2018 geomagnetic storm appeared. To confirm and to describe the fractal nature of analysed data, three methods (structure function, SF, Higuchi, HG and detrended fluctuation analysis, DFA), were applied in the context of estimation of the fractal dimension. The obtained results can be summarised as follows:

- the conducted analysis confirms the fractal nature of the particular geomagnetic field components $B_{X}$ and $B_{Y}$ and reveals differences between their complexity;
- the comparison of fractal dimension estimated by using SF, HG and DFA techniques clearly shows that its values depend on the applied methodology, as well as the physical conditions of analysed data;
- SF and HG analysis for stations BEL, HLP, SOD show an increase in fractal dimension of $B_{Y}$ values during the 26 August 2018 geomagnetic storm;
- DFA reveals a decrease in fractal dimension for both $B_{X}$ and $B_{Y}$ when the main phase of the storm appears;
- the fractal dimension of the Sym-H index, independent of the applied technique, decreases during the geomagnetic storm;
- the appearance of IP, CME, CIR or HSS seems to have significant influence on the fractal dimension values (their growth or decline) of each horizontal geomagnetic magnetic field component, not straightforwardly visible in the raw $B_{X}$ and $B_{Y}$ data.

Summarizing, the performed analysis suggests that the fractal dimension determined by selected methods can support the discrimination of the different states of the local geomagnetic field fluctuations and their deeper characterization. In particular, one can see that the fractal dimension estimated by using SF, HG and DFA techniques presents some changes during physical conditions related to IP shock, CME, CIR or HSS passage during the storm. Especially, just after IP shock occurrence, we see a decrease in $D_{F}$ for all stations, and at the beginning of CME, a rapid and short-lasting reduction of $D_{F}$ is also observed for DFA. We can speculate that these observations can be partially explained by the expected change of the nature of scaling of the geomagnetic field during the mentioned events. Further systematic studies are needed and can bring support for the identification of periods with different physical mechanisms responsible for the change of configuration and dynamics of the geomagnetic field during the storm.

Moreover, we see that the DFA results for the three mid-latitude stations, as well as all method-independent results for the Sym-H index, confirm the findings from previous works (discussed in the Introduction) that the fractal dimension shows significant variation but in general decreases during geomagnetic storms. The comparison of the results obtained for the horizontal geomagnetic field and the Sym-H index, which is not straightforward, reveals some discrepancies. The source of these differences is that all the stations used as a base for the Sym-H index are located at much lower latitudes than the stations used in our analysis. Hence, the dynamics of the changes might be affected by, e.g., substorms, whose nature is different from the storm, as discussed in details in [23].

Further systematic studies need to be carried out on geomagnetic field data from other world observatories. Moreover, various classes of geomagnetic storms should still be considered to confirm the observations and conclusions made for this G3-type storm. We also plan to carry out a systematic comparison of the fractal nature of the geomagnetic components with the geoelectric field computed by using the 1-D layered conductivity model, in particular to verify whether and how the fractal nature is transferred between these two fields. Finally, we are convinced that the application of the multifractal description e.g., [64,65,66], can lead to new insights into the complex nature of geomagnetic field fluctuations during various space weather conditions.

## Figures and Tables

**Table 1:** The stations, whose data have been used in the computations.

Code	Name	Geogr. Lat	Geogr. Lon	CGM Lat	CGM Lon
		[${}^{\circ }$]	[${}^{\circ }$]	[${}^{\circ }$]	[${}^{\circ }$]
BEL	Belsk	51.84	20.79	47.67	95.81
HLP	Hel	54.70	18.81	50.70	95.21
SOD	Sodankylä	67.37	26.63	63.92	107.26
HRN	Hornsund	77.00	15.55	74.13	109.59

**Table 2:** Root Mean Square Error (RMSE) as an average distance between the values of $D_{F}$ determined using SF, HG and DFA methods and the theoretical values of $D_{F}$. Three parametric fractal functions WCF, TF and FBM were used during the tests.

*N*	RMSE-SF			RMSE-HG			RMSE-DFA		
	WCF	TF	FBM	WCF	TF	FBM	WCF	TF	FBM
128	0.12	0.08	0.10	0.08	0.07	0.07	0.07	0.06	0.16
256	0.08	0.09	0.10	0.05	0.07	0.07	0.03	0.05	0.07
512	0.09	0.06	0.12	0.05	0.06	0.09	0.02	0.04	0.14
1024	0.09	0.06	0.10	0.05	0.06	0.09	0.01	0.03	0.10
2048	0.08	0.06	0.06	0.05	0.06	0.07	0.01	0.03	0.05
4096	0.08	0.06	0.08	0.05	0.06	0.08	0.01	0.03	0.11

**Table 3:** Mean Error (ME) of $D_{F}$ determined using SF, HG and DFA methods for three parametric fractal functions WCF, TF and FBM.

*N*	ME-SF			ME-HG			ME-DFA		
	WCF	TF	FBM	WCF	TF	FBM	WCF	TF	FBM
128	−0.05	−0.03	0.04	−0.02	0.02	0.02	−0.07	−0.04	0.00
256	−0.02	−0.05	0.05	0.02	0.02	0.02	−0.02	−0.02	0.01
512	−0.03	−0.02	0.05	0.01	0.02	0.03	−0.02	−0.01	0.04
1024	−0.03	−0.02	0.03	0.01	0.02	0.00	−0.01	0.00	0.02
2048	−0.02	−0.02	0.02	0.01	0.03	0.00	−0.01	0.01	0.00
4096	−0.02	−0.02	0.04	0.01	0.03	0.03	−0.01	0.01	0.04

## Data Availability

Data is available at https://www.intermagnet.org/, http://wdc.kugi.kyoto-u.ac.jp/ (accessed on 29 October 2021).
